# Health literacy in primary care users from six Spanish autonomous communities: a multicenter study of territorial variation

**DOI:** 10.1016/j.pmedr.2026.103677

**Published:** 2026-10-23

**Authors:** David García-García, Andrea González-Ayala, Cristina María Lozano-Hernández, Martín Rodríguez-Álvaro, Pedro Ruymán Brito-Brito, Macarena Chacón-Docampo, María Gil-Marchena, Francisco Javier Pérez-Rivas

**Affiliations:** a Medical Oncology Clinical Trials Unit, Hospital Universitario Marqués de Valdecilla, Instituto de Investigación Marqués de Valdecilla (IDIVAL), Santander, Spain; b UCM Research Group "Public Health – Lifestyles, Nursing Methodology and Care in the Community Setting", Department of Nursing, Faculty of Nursing, Physiotherapy and Podiatry, Complutense University of Madrid, 28040 Madrid, Spain; c Family and Community Care Specialist Nurse, Nursing Manager at José Luis Santamaría Health Centre, Burgos, Spain; d Department of Nursing, Physiotherapy and Occupational Therapy, Faculty of Nursing, University of Castilla-La Mancha, Albacete, Spain; e Institute for Health Research of Castilla-La Mancha (IDISCAM), Albacete, Spain; f Research Network on Chronicity, Primary Care and Health Promotion -RICAPPS-(RICORS), ISCIII, Spain; g Primary Health Care, La Palma Health Services, The Canary Islands Health Service, 38713 Breña Alta, Spain; h Nursing Department, Healthcare Sciences School, University of La Laguna, 38200 Santa Cruz de Tenerife, Spain; i Primary Health Care, Tenerife Primary Health Care Services, The Canary Islands Health Service, 38005 Tenerife, Spain; j Nursing Department, Faculty of Nursing, University of La Laguna, 38200 Santa Cruz de Tenerife, Spain; k INVESTIC Translational Care Research Group, Galicia Sur Health Research Institute (IIS Galicia Sur), SERGAS-UVIGO, Spain; l Meixoeiro University School of Nursing, UVIGO, 36214 Vigo, Galicia, Spain; m Care Research Unit, Vigo Health Area, Álvaro Cunqueiro Hospital, SERGAS, Spain; n Family and Community Care Specialist Nurse. Manager of San Roque Health Centre. Badajoz. Extremadura Health Service, Spain; o Nursing Department, Faculty of Nursing, Physiotherapy and Podiatry, Complutense University of Madrid, Spain; p Health Research Institute 12 de Octubre Hospital (Imas12), 28041 Madrid, Spain

**Keywords:** Health literacy, Primary health care, Health disparities, Social determinants of health, Vulnerable populations, Spain

## Abstract

**Objective:**

Multicenter evidence comparing health literacy across Spanish autonomous communities in primary care remains scarce. We aimed to describe health literacy among adults attending scheduled nursing consultations and explore sociodemographic, clinical, and territorial differences.

**Methods:**

Multicenter cross-sectional study in primary care health centers of six Spanish autonomous communities, conducted between June 2025 and January 2026. Adults aged 18–75 years (*n* = 980) completed the 16-item European Health Literacy Survey Questionnaire, Ponte a 100 lifestyle questionnaire, and 13-item Orientation to Life Questionnaire. Non-parametric tests and multiple linear regression were used.

**Results:**

Among 920 participants with valid scores, 62.50% presented sufficient health literacy and 37.50% problematic or inadequate health literacy. Health literacy was largely homogeneous across sociodemographic groups. Older age was weakly associated with lower health literacy, whereas chronic disease and sense of coherence were associated with higher health literacy; lifestyle was not retained. Participants from Castilla-La Mancha and Castilla y León showed slightly lower adjusted health literacy scores. The model explained 9.50% of health literacy variability.

**Conclusions:**

Sufficient health literacy predominated, but more than one third of participants had limited health literacy, and territorial differences were small. Contextual and healthcare-related factors may shape health literacy beyond individual characteristics.

## Highlights

• Health literacy was generally adequate among primary care users.
• Health literacy was broadly similar across sociodemographic groups.
• Older age was associated with lower health literacy.
• Small health literacy differences were observed across Spanish regions.
• Individual-level factors explained little variability in health literacy.

## 1 Introduction

Health literacy encompasses the ability to access, understand, appraise, and apply health information (Tamayo-Fonseca et al., 2023; Sørensen et al., 2013). It also depends on healthcare organizations' responsiveness and the social contexts shaping health decisions (Sørensen et al., 2012). This study measured personal health literacy using the 16-item European Health Literacy Survey Questionnaire (HLS-EU-Q16). Limited health literacy is associated with difficulties navigating services, poorer adherence, and adverse outcomes, making it an important social determinant of health and health-promotion priority (Tamayo-Fonseca et al., 2023; Estrela et al., 2025; Nutbeam and Lloyd, 2021; World Health Organization, 2017). The European Health Literacy Project (HLS-EU) found limited or problematic health literacy in almost half of adults across eight countries, with substantial national variation (Falcón and Luna, 2012). This distinction is relevant to primary care, where the accessibility of information and the clarity of communication can influence patients' ability to make informed decisions. It also helps explain why individual scores may not fully capture organizational barriers to health literacy. Reporting the median alongside the categorical distribution is important because a score at the lower boundary of the sufficient category does not imply uniformly high literacy across the sample. The three-category presentation provides complementary information on the proportion of patients potentially requiring additional communication support.

Health literacy reflects social inequalities (Tamayo-Fonseca et al., 2023; Sørensen et al., 2015; Garcia-Codina et al., 2019). In Spain, limited health literacy particularly affects older adults, socioeconomically disadvantaged groups, and people with complex chronic conditions. Estimates range from approximately 15% inadequate health literacy in Catalonia's general population (Garcia-Codina et al., 2019) to nearly 80% among older primary care patients with complex chronic conditions (Santesmases-Masana et al., 2017), highlighting inequalities obscured by population averages (Garcia-Codina et al., 2019; Santesmases-Masana et al., 2017; Stormacq et al., 2019; Mantwill et al., 2015).

Older age, lower education, and socioeconomic disadvantage are associated with lower health literacy, difficulties managing chronic conditions, and less healthy lifestyles (Estrela et al., 2025; García-García et al., 2023; Sandri et al., 2025). From a salutogenic perspective, health literacy and sense of coherence (SOC) are complementary assets: SOC supports the appraisal and motivation needed to translate information into health-promoting action (Saboga-Nunes et al., 2019). SOC may also be lower in disadvantaged populations (Escobar-Segovia et al., 2024; Giglio et al., 2015). In primary care, low health literacy has coincided with lower SOC, and both constructs have improved following health-promotion interventions, supporting their joint assessment (Löfgren et al., 2023). The relationship between these resources is particularly relevant for nursing consultations, which often combine health education with support for self-management. Patients may understand the information provided yet encounter practical difficulties in applying recommendations. Considering SOC alongside health literacy therefore offers a broader perspective on how individuals interpret health challenges and mobilize available resources. This conceptual distinction does not imply that either construct independently determines health behavior.

Territorial variation may further shape health literacy in Spain's decentralized National Health System, where health education, prevention, and clinical support differ across autonomous communities (Tamayo-Fonseca et al., 2023; Juvinyà-Canal et al., 2018). Regional policies and access to information may contribute to inequalities, but evidence remains fragmented across local studies using heterogeneous instruments (Tamayo-Fonseca et al., 2023; Sørensen et al., 2013; Santesmases-Masana et al., 2017; Navas-Echazarreta et al., 2024). Nevertheless, observed geographic differences cannot automatically be attributed to regional policies or service organization. The composition of the populations attending participating centers, their socioeconomic circumstances, and local access to health information may also contribute. Studies comparing territories therefore require careful attention to recruitment methods and the extent to which the samples represent the wider populations of each autonomous community.

Primary care offers an opportunity to examine health literacy across diverse social and clinical groups. This study aimed to describe health literacy and explore sociodemographic, clinical, and territorial differences among primary care users in six Spanish autonomous communities.

## 2 Methods

### 2.1 Study design and population

This multicenter cross-sectional study was conducted in public primary care centers in Canarias, Castilla y León, Castilla-La Mancha, Extremadura, Galicia, and Madrid. Centers were recruited by open invitation and professional self-selection (convenience sampling); neither centers nor regions were selected probabilistically. Nurses conducted assessments during routine consultations without additional fieldwork funding. Participating centers provided chronic disease follow-up, prevention, and health education within the Spanish National Health System.

Adults aged 18–75 years attending routine nursing appointments were eligible after written informed consent. The upper age limit was predefined because HLS-EU-Q16 appraisal items require cognitive functions that may vary with age; age-related differential item functioning and associations with cognitive decline have been reported (Rouquette et al., 2018; Geboers et al., 2018). Assessment beyond 75 years could have required cognitive screening or assisted administration, infeasible during routine appointments. The Spanish HLS-EU-Q16 has adequate reliability and a unifactorial structure (Nolasco et al., 2020). This restriction was adopted before recruitment rather than in response to the observed results. Its purpose was to support comparable self-reported responses under the practical conditions of routine nursing care. It should not be interpreted as indicating that the instrument is unsuitable for all adults over 75 years; rather, extending the study to older age groups would have required additional procedures that were outside the scope of this protocol.

Exclusion criteria included acute medical conditions, palliative care, institutionalization, bedridden status, or inability to understand the study procedures due to cognitive or language limitations.

The minimum target was 156 participants per region (936 overall), based on a previously reported health literacy–lifestyle correlation (*r* = 0.22), two-sided α = 0.05, and 80% power (García-García et al., 2023). This calculation did not address multivariable modeling or regional pairwise comparisons; territorial findings are exploratory, and nonsignificance does not establish equivalence. Between June 2025 and January 2026, 980 participants were recruited: Canarias 157, Castilla y León 156, Castilla-La Mancha 168, Extremadura 145, Galicia 158, and Madrid 196. Extremadura reached 92.95% of its regional target, warranting caution. Of 1020 approached, 18 were ineligible and 980/1002 eligible patients participated (97.80%). The regional sample targets were intended to support the planned association analysis, not to establish equivalent precision for every territorial comparison. Consequently, the absence of statistically significant differences between two communities should not be interpreted as proof that their underlying health literacy distributions are the same. This consideration is especially relevant where recruitment fell short of the target or where subgroup sizes were small.

Sampling proceeded in two stages. Regional investigators recruited volunteer centers and nurses, seeking urban and rural settings; at least 10 professionals participated per community. Within each nurse's scheduled appointments, eligible patients were selected using random-number tables. Thus, centers were selected nonprobabilistically but patients probabilistically. Trained nurses used standardized procedures and Spanish-language validated questionnaires administered through Google Forms. Recruitment continued until regional targets or the study period ended. Data were pseudonymized with unique codes and centrally checked for completeness and consistency. Although this procedure introduced an element of random selection at the patient level, it did not eliminate the potential for selection bias arising from the voluntary participation of centers and nursing professionals. Accordingly, the sample should not be interpreted as nationally representative.

### 2.2 Measures

Health literacy was the primary outcome variable. Lifestyle, sense of coherence, and sociodemographic and health-related characteristics were included as explanatory variables.

Collected variables included age, sex, country of birth, place of residence, living arrangement, educational level, occupation, monthly income, self-perceived health, weekly exposure to natural environments (≥2 vs. <2 h/week), and the presence of chronic disease. Self-perceived health status was assessed using a single question with five response categories: very poor, poor, fair, good, and very good. The presence of chronic disease was self-reported by participants.

Health literacy was assessed using the HLS-EU-Q16. The instrument provides a total score ranging from 0 to 16, with higher scores indicating better health literacy. Scores were categorized as inadequate (0–8), problematic (9–12), and sufficient (13–16). The Spanish version has demonstrated adequate psychometric properties (Nolasco et al., 2020). Scores were calculated for participants answering at least 14 items; 60/980 (6.12%) had three or more missing items and no valid score. Consequently, analyses involving health literacy included the 920 participants with a valid score.

Lifestyle was assessed using the validated 33-item Ponte a 100 questionnaire, which provides a global score ranging from 0 to 100, with higher scores indicating healthier lifestyles (Pérez-Rivas et al., 2023).

Sense of coherence (SOC) was assessed using the validated 13-item Orientation to Life Questionnaire (OLQ-13), which yields scores ranging from 13 to 91, with higher values indicating stronger SOC (Eriksson and Lindström, 2005; Antonovsky, 1993; Virués-Ortega et al., 2007; Vega Martínez et al., 2019).

### 2.3 Statistical analysis

Normality was assessed using the Kolmogorov–Smirnov test and graphical methods. Because variables were not normally distributed, non-parametric analyses were performed. Continuous variables are presented as medians and interquartile ranges (IQR), whereas categorical variables are reported as frequencies and percentages.

Associations between health literacy scores and sociodemographic or clinical variables were evaluated using the Mann–Whitney *U* test (effect size *r*) or the Kruskal–Wallis test (effect size η^2^), as appropriate. Health literacy categories (inadequate, problematic, and sufficient) were compared using χ^2^ or Fisher's exact test when expected cell frequencies were low.

Multiple linear regression examined adjusted associations with health literacy. Candidate variables included sociodemographic factors, health indicators, lifestyle, SOC, and autonomous community (Madrid reference). Forward stepwise selection considered all candidates regardless of bivariate significance, using *p* ≤ 0.05 for entry. Regional coefficients were therefore adjusted for other retained predictors. Linear regression was supported by the sample size (*n* = 920) and normally distributed residuals; residual diagnostics and variance inflation factors were examined. Kruskal–Wallis tests with Bonferroni-adjusted post hoc comparisons assessed unadjusted territorial differences. Analyses used IBM SPSS Statistics version 30, with *p* < 0.05 considered significant. The regression coefficients were interpreted as adjusted associations rather than causal effects, consistent with the cross-sectional design. Although residual diagnostics supported the fitted model, the analysis did not incorporate the clustering of participants within nurses or centers. This limitation is relevant to the precision of estimated associations and was considered when interpreting territorial differences. The model was intended to identify exploratory correlates of health literacy rather than to provide an individual-level prediction tool.

### 2.4 Ethics approval and consent to participate

The study was conducted in accordance with the Declaration of Helsinki and current Spanish regulations. All participants provided written informed consent prior to participation. Data were pseudonymized using unique identification codes and handled in compliance with Regulation (EU) 2016/679 and Organic Law 3/2018 on Personal Data Protection.

The study was approved by the Research Ethics Committee on Medicines of Hospital Universitario Ramón y Cajal (reference number: 20250012 – ESTILVID) and by the Central Research Commission of the Madrid Primary Care Assistance Management (reference number: 20250012).

## 3 Results

### 3.1 Sample description

The study included 980 adults from Madrid (*n* = 196), Castilla-La Mancha (*n* = 168), Galicia (*n* = 158), Canarias (*n* = 157), Castilla y León (*n* = 156), and Extremadura (*n* = 145). Regional median ages ranged from 56.00 to 59.00 years (Table 1).

**Table 1:** Sociodemographic and health-related characteristics of adults aged 18–75 years attending scheduled nursing consultations in primary care health centers, by autonomous community, Spain, June 2025–January 2026 (*n* = 980).

Variables	Categories	Canarias n (%)	Castilla y León n (%)	Castilla-La Mancha n (%)	Extremadura n (%)	Galicia n (%)	Madrid n (%)
Age	Median (interquartile range)	57.00 (19.00)	58.00 (20.00)	59.00 (19.00)	58.00 (28.00)	56.00 (21.00)	57.00 (19.00)
Sex	Female	90 (57.32)	82 (52.56)	104 (61.90)	90 (62.07)	89 (56.33)	104 (53.06)
Education level	Low educational level	47 (29.94)	32 (20.51)	73 (43.45)	40 (27.59)	46 (29.11)	24 (12.24)
	High school or Professional training	45 (28.66)	47 (30.13)	45 (26.79)	31 (21.38)	48 (30.38)	61 (31.12)
	University studies	27 (17.20)	47 (30.13)	29 (17.26)	38 (26.21)	31 (19.62)	85 (43.37)
Country of birth	Foreign-born	25 (15.92)	7 (4.49)	10 (5.95)	0 (0.00)	12 (7.59)	19 (9.69)
Living arrangement	Living with a partner	87 (55.41)	95 (60.90)	104 (61.90)	96 (66.21)	98 (62.03)	124 (63.27)
Occupation	Salaried worker	73 (46.50)	75 (48.08)	69 (41.07)	61 (42.07)	55 (34.81)	92 (46.94)
	Retired/pensioner	56 (35.67)	52 (33.33)	61 (36.31)	54 (37.24)	63 (39.87)	67 (34.18)
Place of residence	Rural	90 (57.32)	16 (10.26)	7 (4.17)	21 (14.48)	109 (68.99)	11 (5.61)
Monthly income	≤€1000	57 (36.31)	15 (9.62)	32 (19.05)	21 (14.48)	31 (19.62)	30 (15.31)
	€1001–2500	80 (50.96)	105 (67.31)	94 (55.95)	78 (53.79)	94 (59.49)	118 (60.20)
	>€2500	4 (2.55)	15 (9.62)	11 (6.55)	6 (4.14)	8 (5.06)	25 (12.76)
Self-perceived health	Good/very good	93 (59.24)	120 (76.92)	96 (57.14)	96 (66.21)	87 (55.06)	131 (66.84)
Nature connection	≥2 h/week	96 (61.15)	90 (57.69)	83 (49.40)	66 (45.52)	115 (72.78)	120 (61.22)
Chronic disease presence	Yes	111 (70.70)	104 (66.67)	131 (77.98)	116 (80.00)	124 (78.48)	144 (73.47)

Values are n (%) unless otherwise indicated. Percentages are calculated over the total number of participants in each autonomous community. The interquartile range is expressed as the difference between the 75th and 25th percentiles. For living arrangement, occupation, and monthly income, only the most frequent categories are shown; the remaining categories (living arrangement: with children, with parents or other relatives, with roommates, alone, other; occupation: self-employed, unemployed, unpaid domestic work, student; monthly income: no income, does not know or does not answer) are presented in Table 3.

^a^Includes illiterate, incomplete primary education, and completed primary education.

Participants were predominantly female and Spanish-born; high school or professional training, monthly incomes of €1001–2500, living with a partner, and salaried work or retirement were common (Table 1).

Territorial differences were observed across the participating regions. Madrid showed the highest proportion of participants with university education (43.37%) and monthly incomes above €2500 (12.76%). In contrast, Castilla-La Mancha had the highest proportion of participants with low educational attainment (43.45%), whereas Canarias had the highest percentages of foreign-born individuals (15.92%) and low-income earners (36.31%). Rural residence was most frequent in Galicia (68.99%) and Canarias (57.32%), whereas urban residence predominated in Madrid, Castilla-La Mancha, and Castilla y León (Table 1).

The prevalence of chronic disease was high across all regions, ranging from 66.67% in Castilla y León to 80.00% in Extremadura. Hypertension, diabetes mellitus, and obesity were the most frequently reported chronic conditions (Fig. 1).

**Fig. 1:**
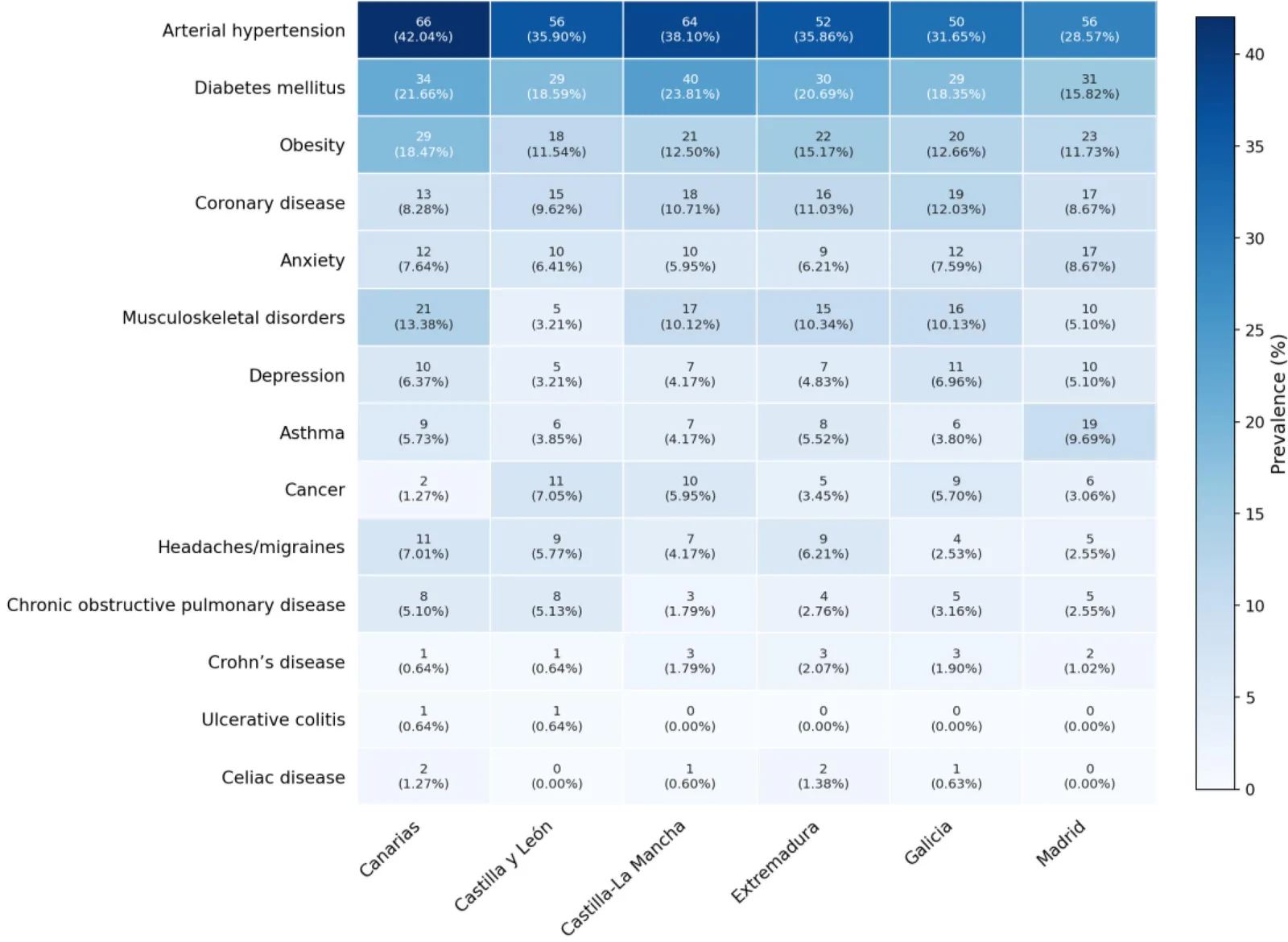
Prevalence of chronic conditions among adults aged 18–75 years attending scheduled nursing consultations in primary care health centers, by autonomous community, Spain, June 2025–January 2026 (*n* = 980). Values are n (%). Percentages were calculated over all participants in each autonomous community: Canarias (*n* = 157), Castilla y León (*n* = 156), Castilla-La Mancha (*n* = 168), Extremadura (*n* = 145), Galicia (*n* = 158), and Madrid (*n* = 196). Because participants could report more than one chronic condition, categories are not mutually exclusive and percentages may sum to more than 100%. Color intensity represents prevalence (%). COPD, chronic obstructive pulmonary disease.

### 3.2 Health literacy levels according to sociodemographic and health-related variables

The overall median HLS-EU-Q16 score was 13.00 (IQR = 4.00), the threshold for sufficient health literacy; over one third had problematic or inadequate health literacy (Table 3). Regional medians ranged from 13.00 to 14.00 (Table 2).

**Table 2:** Health literacy scores by sociodemographic and health-related characteristics among adults aged 18–75 years attending scheduled nursing consultations in primary care health centers in six Spanish autonomous communities, June 2025–January 2026 (*n* = 920).

Variables		Health literacy score (Median [interquartile range])	*P* value	Effect size (r or η^2^)
Overall health literacy score		13.00 (4.00)		

Sex				
	Male	13.00 (4.00)	0.38	*r* = −0.03
	Female	14.00 (4.00)		
	Place of Residence			
	Urban	13.00 (4.00)	0.84	*r* = −0.01
	Rural	13.00 (5.00)		
	Country of Birth			
	Spain	13.00 (4.00)	0.40	*r* = −0.03
	Foreign-born	14.00 (4.00)		
	Living Arrangement			
	With partner (married or unmarried)	13.00 (4.00)	0.18	η^2^ < 0.01
	With children	13.00 (5.00)		
	With parents or other relatives	14.00 (3.00)		
	With roommates	15.00 (3.00)		
	Alone	13.00 (4.00)		
	Other	15.50 (4.00)		

Occupation				
	Salaried worker	13.00 (3.00)	0.12	η^2^ < 0.01
	Self-employed	13.00 (4.00)		
	Unemployed	14.00 (4.00)		
	Retired/pensioner	13.00 (4.00)		
	Unpaid domestic work	14.00 (5.00)		
	Student	15.00 (4.00)		

Education level				
	Illiterate or incomplete primary education	13.00 (3.00)	0.31	η^2^ < 0.01
	Primary education	13.00 (4.00)		
	Secondary education	14.00 (4.00)		
	High school or Professional training	13.00 (4.00)		
	University studies	14.00 (3.00)		

Monthly income				
	No income	14.00 (5.00)	0.60	η^2^ < 0.01
	≤1000€	13.00 (5.00)		
	1001–2500€	13.00 (4.00)		
	>2501€	14.00 (3.00)		
	Don't know/no answer	13.00 (4.00)		

Self-perceived Health Status				
	Very poor	15.50 (3.00)	0.44	η^2^ < 0.01
	Poor	13.00 (5.00)		
	Fair	14.00 (3.00)		
	Good	13.00 (4.00)		
	Very good	14.00 (3.00)		

Nature connection				
	< 2 h/week	13.00 (4.00)	0.32	r = −0.03
	≥ 2 h/week	13.00 (3.00)		

Chronic Disease				
	Yes	14.00 (4.00)	0.33	r = −0.03
	No	13.00 (4.00)		

Autonomous Community				
	Canarias	14.00 (4.00)	0.03	η^2^ < 0.01
	Castilla y León	13.00 (4.00)		
	Castilla-La Mancha	13.00 (5.00)		
	Extremadura	13.00 (4.00)		
	Galicia	14.00 (4.00)		
	Madrid	14.00 (3.00)		

Health literacy was measured with the 16-item European Health Literacy Survey Questionnaire (HLS-EU-Q16; score range 0–16). Of the 980 participants, 60 (6.12%) had no valid total score because they answered fewer than 14 of the 16 items and were excluded. The interquartile range is expressed as the difference between the 75th and 25th percentiles. *P*-values were obtained from the Mann–Whitney *U* test (two categories) or the Kruskal–Wallis test (more than two categories). Effect sizes: r = Mann–Whitney effect size; η^2^ = Kruskal–Wallis eta squared.

Age showed a weak but statistically significant negative correlation with health literacy (ρ = −0.10, *p* < 0.01), indicating slightly lower health literacy scores among older participants.

Health literacy scores differed only by autonomous community (*p* = 0.03), with a very small effect (η^2^ < 0.01). Post hoc comparisons showed higher scores in Madrid than Castilla y León (adjusted *p* = 0.02); other pairwise contrasts were nonsignificant. No significant differences were found for the other sociodemographic or health-related characteristics (Table 2).

### 3.3 Health literacy profiles according to sociodemographic and health-related variables

Overall, among the 920 participants with a valid HLS-EU-Q16 score, most participants presented sufficient health literacy (*n* = 575, 62.50%), whereas 278 (30.22%) showed problematic health literacy and only 67 (7.28%) presented inadequate health literacy (Table 3).

**Table 3:** Distribution of health literacy levels by sociodemographic and health-related characteristics among adults aged 18–75 years attending scheduled nursing consultations in primary care health centers in six Spanish autonomous communities, June 2025–January 2026 (*n* = 920).

Variables	Categories		Health Literacy Categories			χ^2^ or Fisher's exact test	*p*-Value
			Inadequate	Problematic	Sufficient		
Overall			67 (7.28%)	278 (30.22%)	575 (62.50%)		
Sex	Male		29 (7.40%)	118 (30.10%)	245 (62.50%)	ꭓ^2^ = 0.02	0.99
	Female		38 (7.20%)	160 (30.30%)	330 (62.50%)		
Education level	Illiterate or incomplete primary education		5 (12.50%)	13 (32.50%)	22 (55.00%)	ꭓ^2^ = 9.12	0.33
	Primary education		18 (8.78%)	71 (34.63%)	116 (56.59%)		
	Secondary education		13 (7.78%)	46 (27.54%)	108 (64.67%)		
	High school or professional training		20 (7.60%)	77 (29.28%)	166 (63.12%)		
	University studies		11 (4.49%)	71 (28.98%)	163 (66.53%)		
Country of Birth	Spanish		61 (7.18%)	261 (30.74%)	527 (62.07%)	ꭓ^2^ = 1.47	0.48
	Foreign		6 (8.45%)	17 (23.94%)	48 (67.61%)		
Living arrangement	With partner (married or unmarried)		41 (7.28%)	178 (31.62%)	344 (61.10%)	ꭓ^2^ = 8.76	0.56
	With children		5 (6.76%)	22 (29.73%)	47 (63.51%)		
	With parents or other relatives		5 (4.50%)	31 (27.93%)	75 (67.57%)		
	With roommates		1 (6.25%)	2 (12.50%)	13 (81.25%)		
	Alone		12 (8.70%)	42 (30.43%)	84 (60.87%)		
	Other		3 (16.67%)	3 (16.67%)	12 (66.67%)		
Occupation	Salaried worker		23 (5.76%)	116 (29.07%)	260 (65.16%)	ꭓ^2^ = 6.37	0.78
	Self-employed		6 (10.17%)	18 (30.51%)	35 (59.32%)		
	Unemployed		4 (6.15%)	16 (24.62%)	45 (69.23%)		
	Retired/pensioner		29 (8.76%)	107 (32.33%)	195 (58.91%)		
	Unpaid domestic work		3 (8.33%)	12 (33.33%)	21 (58.33%)		
	Student		2 (6.67%)	9 (30.00%)	19 (63.33%)		
Place of Residence	Urban		45 (6.61%)	201 (29.52%)	435 (63.88%)	ꭓ^2^ = 2.86	0.24
	Rural		22 (9.21%)	77 (32.22%)	140 (58.58%)		
Monthly income	No income		4 (6.35%)	19 (30.16%)	40 (63.49%)	ꭓ^2^ = 8.05	0.43
	≤1000€		13 (7.51%)	56 (32.37%)	104 (60.12%)		
	1001 - 2500€		34 (6.36%)	164 (30.65%)	337 (62.99%)		
	>2501€		4 (6.15%)	18 (27.69%)	43 (66.15%)		
	Doesn't know/doesn't answer		12 (14.29%)	21 (25.00%)	51 (60.71%)		
Self-perceived Health Status	Very poor		0 (0.00%)	1 (16.67%)	5 (83.33%)	ꭓ^2^ = 7.25	0.53
	Poor		3 (9.09%)	13 (39.39%)	17 (51.52%)		
	Fair		17 (5.84%)	81 (27.84%)	193 (66.32%)		
	Good		41 (8.12%)	161 (31.88%)	303 (60.00%)		
	Very good		6 (7.06%)	22 (25.88%)	57 (67.06%)		
Nature Connection	<2 h/week		27 (7.03%)	123 (32.03%)	234 (60.94%)	ꭓ^2^ = 1.03	0.60
	≥2 h/week		40 (7.46%)	155 (28.92%)	341 (63.62%)		
Chronic Disease	Yes		51 (7.47%)	202 (29.58%)	430 (62.96%)	ꭓ^2^ = 0.57	0.75
	No		16 (6.75%)	76 (32.07%)	145 (61.18%)		
Autonomous Community		Canarias	13 (9.09%)	38 (26.57%)	92 (64.34%)	ꭓ^2^ = 30.35	<0.01
		Castilla y León	13 (8.55%)	56 (36.84%)	83 (54.61%)		
		Castilla-La Mancha	21 (13.29%)	36 (22.78%)	101 (63.92%)		
		Extremadura	7 (5.11%)	51 (37.23%)	79 (57.66%)		
		Galicia	10 (6.85%)	46 (31.51%)	90 (61.64%)		
		Madrid	3 (1.63%)	51 (27.72%)	130 (70.65%)		

Percentages are row percentages. Health literacy was measured with the 16-item European Health Literacy Survey Questionnaire (HLS-EU-Q16) and categorized as inadequate (0–8), problematic (9–12), or sufficient (13–16). Of the 980 participants, 60 (6.12%) had no valid total score because they answered fewer than 14 of the 16 items and were excluded. *P*-values were obtained from the χ^2^ test or Fisher's exact test, as appropriate.

Health literacy categories did not differ significantly across the other sociodemographic or health-related characteristics assessed.

Autonomous community was the only variable significantly associated with health literacy distribution (χ^2^ = 30.35, *p* < 0.01). Madrid presented the highest proportion of participants with sufficient health literacy (70.65%) and the lowest proportion with inadequate health literacy (1.63%), whereas Castilla-La Mancha showed the highest proportion of inadequate health literacy (13.29%).

### 3.4 Health literacy multiple linear regression model

The multivariable linear regression model explained 9.50% of the variability in health literacy, indicating that individual characteristics accounted for only a small part of the observed differences (Table 4).

**Table 4:** Factors associated with health literacy scores in a multivariable linear regression model among adults aged 18–75 years attending scheduled nursing consultations in primary care health centers in six Spanish autonomous communities, June 2025–January 2026 (*n* = 920).

Predictors	B (95% CI)
Constant	11.40 (9.87, 12.93)
Sense of coherence score^b^	0.06 (0.05, 0.08)
Autonomous Community (reference: Madrid): Castilla-La Mancha	−0.80 (−1.30, −0.30)
Chronic Disease Presence (reference: yes): No	−0.59 (−1.05, −0.12)
Autonomous Community (reference: Madrid): Castilla y León	−0.57 (−1.08, −0.06)
Age^a^ (years)	−0.03 (−0.04, −0.01)

Health literacy was measured with the 16-item European Health Literacy Survey Questionnaire (HLS-EU-Q16; score range 0–16). Predictors were selected by forward stepwise selection (entry criterion *p* ≤ 0.05) from all candidate variables; lifestyle and the remaining sociodemographic and health-related variables did not meet the entry criterion. Model summary: Explained variance (R^2^) = 9.50%; adjusted explained variance (adjusted R^2^) = 9.00%; F(5, 914) = 19.20.

CI, confidence interval.

a Age was modeled as a continuous variable.

b Measured with the 13-item Orientation to Life Questionnaire (OLQ-13; score range 13–91). Autonomous community was entered as dummy variables; only those for Castilla-La Mancha and Castilla y León met the entry criterion.

Older age was independently associated with slightly lower health literacy scores, whereas the presence of chronic disease was associated with higher health literacy. Sense of coherence showed a weak positive association with health literacy, while lifestyle was not significantly associated.

Regarding territorial differences, participants from Castilla-La Mancha and Castilla y León presented slightly lower health literacy scores than those from Madrid after adjustment for individual characteristics.

The model's limited explanatory capacity suggests that contextual and healthcare-system factors not measured here may also contribute to health literacy differences.

## 4 Discussion

Most primary care participants across six Spanish autonomous communities had sufficient health literacy. Sociodemographic differences were generally absent; territorial differences and the association with age were small. The sample's exclusion of adults older than 75 years may have limited detection of vulnerability in older groups.

Sufficient health literacy may reflect the sample's age restriction, predominantly Spanish-born participants, educational and income profiles, and frequent primary care contact among patients with chronic disease. Volunteer-center recruitment and nurse administration may also have introduced selection and social desirability biases. Limited health literacy (37.50%) exceeded estimates for the general populations of Catalonia (15.40%) and Valencia (12.80%) (Tamayo-Fonseca et al., 2023; Garcia-Codina et al., 2019), possibly reflecting differences in age and chronic disease burden. Comparisons with HLS-EU findings, including socioeconomic gradients, require caution because instruments and thresholds differ (Sørensen et al., 2015). The discrepancy with population surveys should therefore be interpreted descriptively rather than as evidence of a true difference in the underlying populations. Primary care users are not a random sample of community residents, and frequent contact with services may change both the information available to patients and their familiarity with healthcare terminology. Moreover, interviewer administration can affect responses differently from self-completed surveys, making direct comparisons across studies difficult.

Territorial differences were small: Madrid scored higher than Castilla y León in unadjusted comparisons, while Castilla-La Mancha and Castilla y León scored slightly lower than Madrid after adjustment. Educational, socioeconomic, or healthcare differences could contribute, but were not measured. Because centers were selected nonprobabilistically, these comparisons are exploratory. Territorial context nevertheless merits consideration in health-literacy strategies (Navas-Echazarreta et al., 2024; Baccolini et al., 2021; Valls Martínez et al., 2022). The findings should not be taken as evidence of differences in regional health-system performance, since organizational characteristics and local service resources were not measured. Further studies could examine whether variation in service accessibility, communication practices, and community resources contributes to these patterns.

Older age was weakly associated with lower health literacy, potentially reflecting cognitive and sensory changes and difficulties navigating increasingly complex healthcare systems (Tamayo-Fonseca et al., 2023; Baccolini et al., 2021; García-García and Pérez-Rivas, 2022). Age-related barriers warrant consideration in primary care interventions.

The model explained only 9.50% of health literacy variability, suggesting that unmeasured contextual and healthcare factors may be important (Sørensen et al., 2013; Stormacq et al., 2019; Levic et al., 2023). Organizational responsiveness—including clear communication, sufficient consultation time, interpretation, and culturally appropriate care—may complement individual competencies (Peprah et al., 2023; Peprah et al., 2024).

Higher health literacy among patients with chronic disease may reflect experiential learning through repeated healthcare contact (Tamayo-Fonseca et al., 2023; Santesmases-Masana et al., 2017; Chauhan et al., 2024). SOC was weakly positively associated with health literacy, whereas lifestyle was not associated after adjustment. Health information alone may not translate into healthier behaviors when time, financial, food-environment, or physical-activity barriers persist (Estrela et al., 2025; García-García et al., 2023; Sandri et al., 2025). Repeated consultations may provide opportunities to clarify recommendations, ask questions, and become familiar with chronic disease management. However, this explanation remains hypothetical because neither the frequency nor the quality of patient–professional interactions was directly evaluated. Likewise, the absence of an independent lifestyle association does not rule out relationships between specific health behaviors and health literacy. The global lifestyle score may obscure variation across individual behaviors, and contextual barriers can constrain behavior even when information is understood.

Strengths include multicenter recruitment, standardized procedures, and validated instruments. However, the cross-sectional design precludes causal inference. Restriction to primary care users aged 18–75 years limits generalizability, especially to older adults. Volunteer recruitment of centers and nonprobabilistic selection of regions may have introduced selection bias and undermine territorial representativeness. Sample size was calculated for associations, not regional contrasts; comparisons are exploratory, particularly in Extremadura, which missed its target. Patients were clustered within nurses and centers, but clustering was not modeled, potentially underestimating standard errors. Routine-appointment recruitment may have favored healthcare-engaged patients, and nurse-administered questionnaires may have induced social desirability. Unmeasured structural and healthcare factors prevent causal attribution of territorial differences. Country of birth alone did not capture migration-related factors such as language proficiency or length of residence. Future studies should use representative territorial samples and multilevel designs incorporating contextual and healthcare-system measures. These limitations are particularly important when interpreting the small regional coefficients, whose statistical significance may be sensitive to assumptions about independence of observations. The participating autonomous communities also represent only the locations where collaborating centers were available, rather than a designed sample of the Spanish healthcare system. Future research should consider recruiting centers through predefined sampling frames and collecting organizational indicators alongside individual measures to distinguish compositional from contextual variation.

## 5 Conclusions

Health literacy was generally sufficient among primary care users in six Spanish autonomous communities, with small territorial differences and a weak negative association with age. The model's limited explanatory capacity suggests that contextual and healthcare-system factors warrant further study. Population-based, territorially responsive strategies should address disadvantaged groups and age-related barriers.

## Availability of data and materials

The datasets used and analyzed during the current study are available from the corresponding author on reasonable request.

## CRediT authorship contribution statement

**David García-García:** Writing – review & editing, Writing – original draft, Methodology, Funding acquisition, Formal analysis, Conceptualization. **Andrea González-Ayala:** Writing – review & editing, Funding acquisition, Data curation. **Cristina María Lozano-Hernández:** Writing – review & editing, Data curation. **Martín Rodríguez-Álvaro:** Writing – review & editing, Data curation. **Pedro Ruymán Brito-Brito:** Writing – review & editing, Data curation. **Macarena Chacón-Docampo:** Writing – review & editing, Data curation. **María Gil-Marchena:** Writing – review & editing, Data curation. **Francisco Javier Pérez-Rivas:** Writing – review & editing, Writing – original draft, Methodology, Funding acquisition, Formal analysis, Conceptualization.

## Ethics declaration

Written informed consent to take part in the study and to publish the article has been obtained from all participants or their legal representatives. The privacy rights of participants have been observed.

This study was performed in compliance with relevant laws, regulatory frameworks and guidelines where the research took place. This study was approved by the Research Ethics Committee on Medicines of Hospital Universitario Ramón y Cajal. (Approval No. 20250012)

## Funding

This work was supported by the Regional Health Management of Castilla y León (Gerencia Regional de Salud de Castilla y León, SACYL), through the 2025 Call for Biomedical, Health Services and Social Health Care Research Projects, under grant GRS 3023/C/2024.

## Declaration of competing interest

The authors declare that they have no known competing financial interests or personal relationships that could have appeared to influence the work reported in this paper.

## Data Availability

Data will be made available on request.
